# Inhibition of calcium-stimulated adenylyl cyclase subtype 1 (AC1) for the treatment of pain and anxiety symptoms in Parkinson’s disease mice model

**DOI:** 10.1177/17448069241266683

**Published:** 2024-07-26

**Authors:** Zhaoxiang Zhou, Qi-Yu Chen, Min Zhuo, Ping-Yi Xu

**Affiliations:** 1 Department of Neurology, 117969 The First Affiliated Hospital of Guangzhou Medical University, Guangzhou, China; 2 Department of Physiology, Faculty of Medicine, 7938 University of Toronto, Toronto, ON, Canada; 3 Department of Exercise & Health Science, Xi’an Physical Education University, Xi’an, China; 4 Zhuomin Institute of Brain Research, Qingdao, China

**Keywords:** Anxiety, adenylyl cyclase subtype 1, NB001, Parkinson’s disease, pain

## Abstract

Pain and anxiety are two common and undertreated non-motor symptoms in Parkinson’s disease (PD), which affect the life quality of PD patients, and the underlying mechanisms remain unclear. As an important subtype of adenylyl cyclases (ACs), adenylyl cyclase subtype 1 (AC1) is critical for the induction of cortical long-term potentiation (LTP) and injury induced synaptic potentiation in the cortical areas including anterior cingulate cortex (ACC) and insular cortex (IC). Genetic deletion of AC1 or pharmacological inhibition of AC1 improved chronic pain and anxiety in different animal models. In this study, we proved the motor deficit, pain and anxiety symptoms of PD in 1-methyl-4-phenyl-1,2,3,6-tetrahydropyridine (MPTP)-treated mice model. As a lead candidate AC1 inhibitor, oral administration (1 dose and seven doses) of NB001 (20 and 40 mg/kg) showed significant analgesic effect in MPTP-treated mice, and the anxiety behavior was also reduced (40 mg/kg). By using genetic knockout mice, we found that AC1 knockout mice showed reduced pain and anxiety symptoms after MPTP administration, but not AC8 knockout mice. In summary, genetic deletion of AC1 or pharmacological inhibition of AC1 improved pain and anxiety symptoms in PD model mice, but didn’t affect motor function. These results suggest that NB001 is a potential drug for the treatment of pain and anxiety symptoms in PD patients by inhibiting AC1 target.

## Introduction

As a chronic and progressive neurodegenerative disease, Parkinson’s disease (PD) has been classically predominantly regarded as a movement disorder.^1,2^ In addition to the motor symptoms, patients living with PD literally suffer from the non-motor symptoms NMSs) of PD, such as, pain and anxiety. Pain is a frequent NMS in PD, contributing significantly to disability and significantly reduced health-related quality of life. It affects 40%–85% of PD patients.^3–5^ As another frequent non-motor symptoms in PD, anxiety also affects approximately 40% PD patients.^6^ Both pain and anxiety have become frequent worsening factors of the disease and is associated with lower quality of life, the underlying mechanisms remain largely unknown. Treatments for pain and anxiety symptoms in PD patients show inconsistent efficacy across clinical trials, largely owing to our limited understanding of the mechanisms.^5^

Previous studies of PD focus on midbrain and brainstem areas, however, the role of cortex in PD get more attention.^7^ As a key cortical region in pain perception and emotional regulation, including acute pain, various chronic pain, anxiety, fear emotion and depression,^8,9^ the anterior cingulate cortex (ACC) in PD patients with pain showed a higher pain activation only in the right ACC, but not prefrontal cortex and insular cortex in a positron emission tomography (PET) study. Moreover, our previous study reported that the ACC was activated bilaterally in 1-methyl-4-phenyl-1,2,3,6-tetrahydropyridine (MPTP)-treated mice with pain and anxiety symptoms.^10^ In the mice model of PD, ACC long-term potentiation (LTP) was occluded and synaptic excitatory transmission of the ACC neurons was enhanced, which suggests that ACC may play important role in PD related pain and anxiety.

Neuronal selective adenylyl cyclase subtype 1 (AC1) has been supposed to be a key intracellular protein in cortical regions, which causes different forms of LTP. Inhibiting the activity of AC1 by selective inhibitor NB001 blocks behavioral sensitization and injury-related anxiety in different animal models of chronic pain, including neuropathic pain, inflammatory pain, cancer pain, visceral pain and migraine.^11–14^ Injury-induced changes in excitatory transmission including postsynaptic and presynaptic changes, have been investigated in cortical areas, including ACC^15,16^ and insular cortex (IC). Considering the enhanced excitatory transmission of the ACC neurons in PD mice model, we therefore infer that AC1 may participate the molecular and synaptic mechanism of PD-related pain and anxiety.

To investigate if inhibiting the activity of AC1 affect the behavioral pain and anxiety symptoms in PD mice model, we carried out behavioral methods and genetic approaches to address these questions. The behavioral effect of NB001 on motor function were also examined.

## Materials and methods

### Animals

Adult male C57BL/6 mice (6–8 weeks old) were purchased from the Beijing Vital River Laboratory Animal Technology Co., Ltd. All mice were randomly housed in corncob-lined plastic cages under an artificial 12 h light/12 h dark cycle (lights on 9 a.m. to 9 p.m.) with enough food and water. AC1 and AC8 knockout mice were bred for several generations on a C57BL/6 background. All animal studies followed the institutional guidelines for animal experiments of Guangzhou Medical University. All procedures were approved by the Institutional Animal Care and Use Committee of Guangzhou Medical University.

### MPTP treatment

Mice were injected intraperitoneally (i.p.) with MPTP (20 mg/kg) or sterile saline solution (four times at 2 h intervals).

### Mechanical withdrawal threshold measurement

The mice paw withdrawal threshold was tested with von Frey filaments (Stoelting; Wood Dale, Illinois). The animals were placed in Lucite cubicles over a wire mesh and acclimated for 30 min before testing. A series of filaments (0.008, 0.02, 0.04, 0.16, 0.4, 0.6, 1, 1.4, 2 g) with various bending forces (according to 0.078, 0.196, 0.392, 1.568, 3.92, 5.88, 9.8, 13.72, 19.6 mN) were applied to the plantar surface of the hindpaw until the mice withdrew from the stimulus. Each filament was applied twice. The lowest force at which a withdrawal response was obtained was then taken as the paw withdrawal threshold.

### Mechanical allodynia

Mice were individually placed in a round, transparent container 20 cm in diameter and were allowed to acclimate for 30 min before testing. Mechanical sensitivity was assessed with a set of von Frey filaments. Based on preliminary experiments that characterized the threshold stimulus in untreated animals, the innocuous 0.04 g filament was used to detect mechanical allodynia.^17,18^ The filament was applied to the point of bending six times to the surfaces of the hindpaws. Positive responses consisted of prolonged hindpaw withdrawal followed by licking or scratching. Mechanical threshold was assessed on the basis of the responsiveness of the hindpaw to the application of von Frey filaments to the point of bending. The filament was applied over the dorsum of the paw while the animal was resting.

### Elevated plus maze

The elevated plus maze (EPM, Med. Associates) consisted of two open arms and two closed arms situated perpendicular to each other. The maze was situated ∼70 cm from the floor. For each test, mice were individually placed in the center square and allowed to move freely for 5 min. The number of entries and time spent in each arm were recorded. A video camera tracking system (Ethovision) was used to generate the traces.

### RotaRod test

To test motor function, we used a RotaRod from Med. Associates. The RotaRod test was performed by measuring the time each animal was able to maintain its balance while walking on a rotating drum. 1 h before testing, animals were trained on the RotaRod at a constant acceleration of 16 r/min until they could stay on for 30 s. For testing, the RotaRod was set to accelerate from 4 to 40 r/min over a 5 min period. Mice were given three trials with a maximum time of 300 s and a 5 min inter-trial rest interval. The latency to fall was taken as a measure of motor function.

### Homecage behaviors

24 h homecage behaviors were tested using AI homecage system (Shanghai Vanbi Intelligent Technology Co., Ltd). The digital video cameras were mounted perpendicular to the cages. The cameras input into a Pelco video processor connected to computers. Video data were analyzed by Tracking Master software (Shanghai Vanbi Intelligent Technology Co., Ltd). During 24 h recording, mice were housed in standard cages on a 12 h light/dark cycle with food and water provided *ad libitum*.^19^

### Data analysis

Results were expressed as mean ± SEM. Statistical comparisons were performed with one-way ANOVA or two-way ANOVA and Students *t* test. In all cases, *p < .05 was considered statistically significant.

## Results

### Motor deficits, pain and anxiety symptoms exhibit in Parkinson’s disease mice model induced by MPTP injection

**Figure 1.**
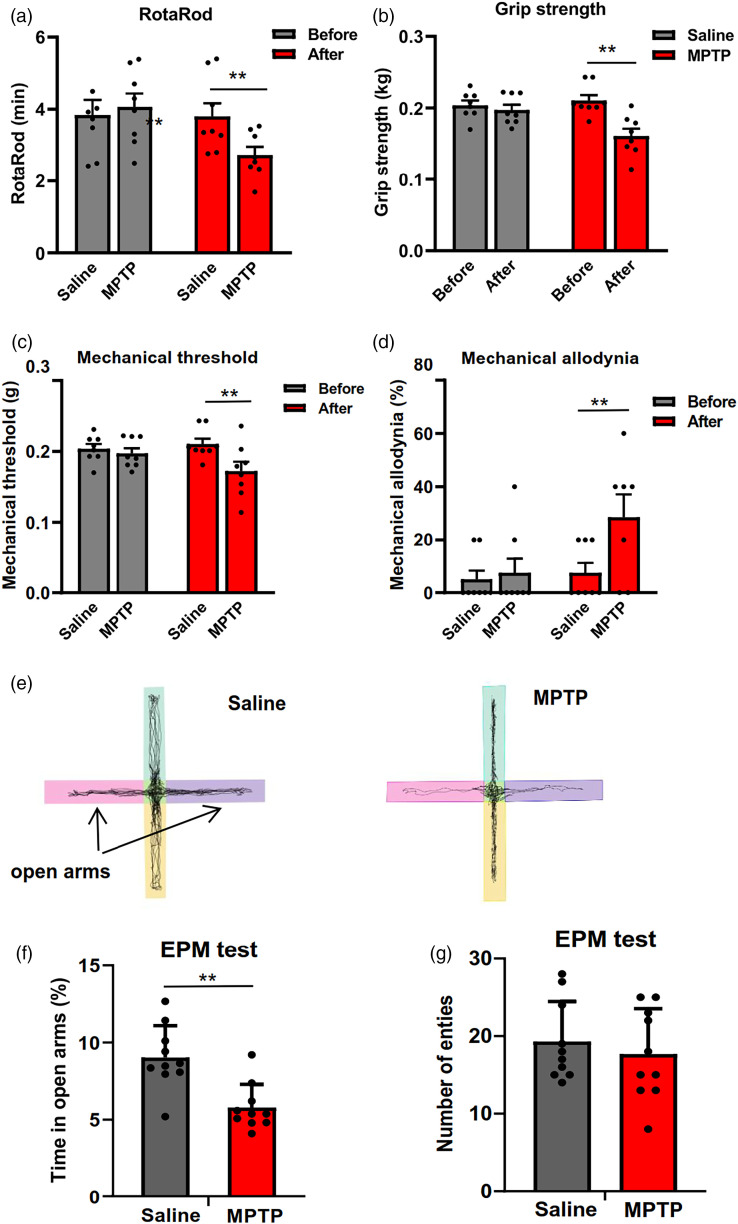
Motor function, pain and anxiety symptoms assessment of MPTP-treated mice. (a) Motor performance of MPTP-treated and 6-OHDA-treated mice were significantly decreased compared with saline-treated mice in the Rotarod test. (b) A severe grip strength deficit was observed in MPTP-treated mice. (c–d) Mechanical threshold was reduced (c) and mechanical allodynia was increased (d) in MPTP-treated mice. (e) Representative traces showing the movement of mice in EPM (f–g) MPTP-treated mice exhibited increased anxiety-related behaviors in EPM (f) and there was no significant difference of entry number in EPM (g). *p < .05, **p < .01.

Since the main symptoms of PD are motor deficits, we performed the RotaRod test and grip strength test, which are commonly used to confirm the phenotypes of PD model animals. Motor function assessed by RotaRod test and grip strength test were significantly declined after MPTP administration (Figure 1(a) and (b)). These results showed MPTP administration impaired the motor function of mice, including muscle capacity, the coordination of movement.

**Figure 2.**
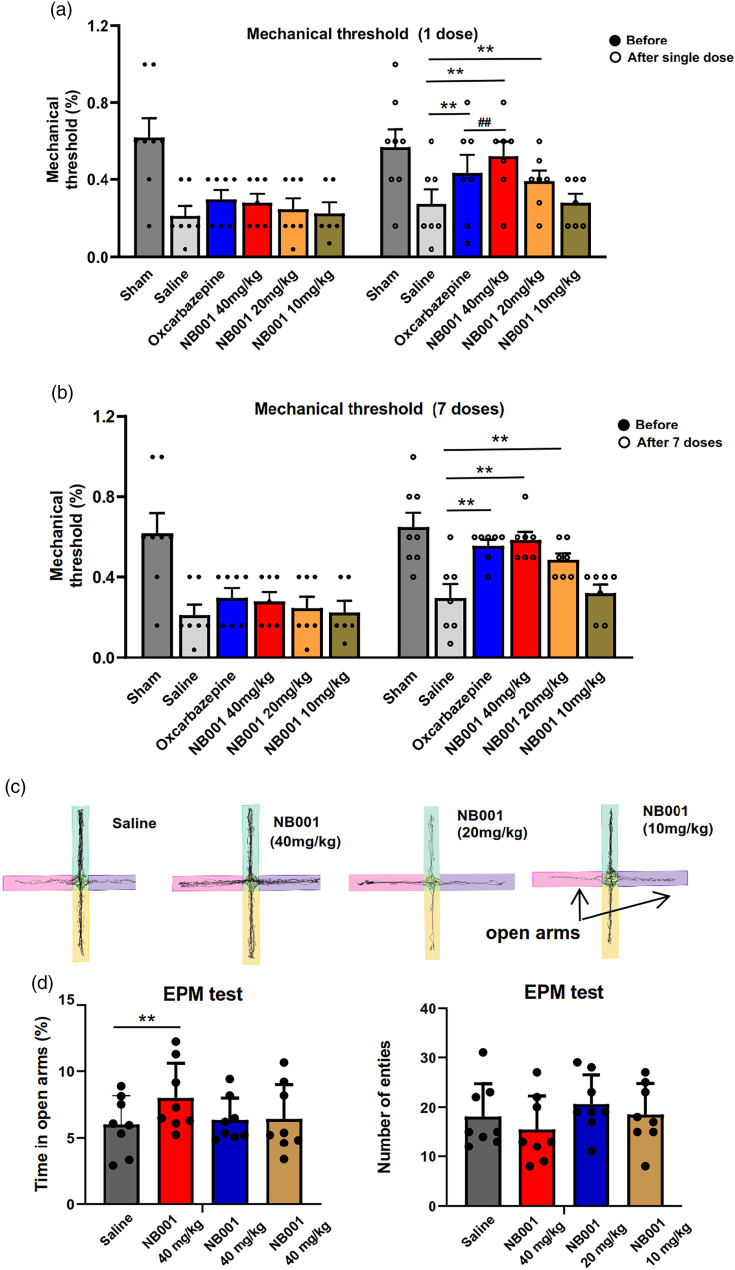
Analgesic and anxiolytic effect of NB001 on MPTP-treated mice. (a) Compared with saline group, oral administration of 40 and 20 mg/kg NB001 (1 dose) increased mechanical threshold of MPTP-treated mice. (b) Oral administration of 40 and 20 mg/kg NB001 (7 dose) increased mechanical threshold of MPTP-treated mice. (c) Representative traces showing the movement of mice in EPM (d–e) After oral administration of 40 mg/kg NB001, MPTP-treated mice exhibited reduced anxiety-related behaviors in EPM (d) and there was no significant difference of entry number in EPM (e). *p < .05, **p < .01.

Both pain and anxiety are prominent non-motor symptoms observed in patients with Parkinson’s disease.^5^ Therefore, we used different nociceptive behavioral tests to measure the mechanical allodynia and hyperalgesia. Compared to saline groups mice, MPTP-treated mice exhibited reduced mechanical threshold and increased responses in mechanical allodynia test (Figure 2(c) and (d)), which is consistent with previous report.^20^ In addition, we performed EPM assess the anxiety behaviors. Decreased time spent in open arms indicated that MPTP-treated mice exhibited increased anxiety-related behaviors (Figure 2(e) and (f)).

Taken together, these results indicate that the MPTP administration induces motor deficits, increased pain and anxiety-related behaviors.

### Oral administration of NB001 improve the pain and anxiety-related behaviors, but not motor function in MPTP-treated mice

With the identification of NB001 as an AC1 inhibitor, we examined the effects of NB001 on behavioral hyperalgesia in the animal model of Parkinson’s disease. Oral administration of NB001 given 45 min before behavioral hyperalgesia testing produced a significant analgesic effect.

After one dose administration, high dose (40 mg/kg) and medium dose (20 mg/kg) of NB001produced a great inhibition of behavioral hyperalgesia, especially high dose, which showed better analgesic effect than oxcarbazepine, an anti-epileptic drug used in pain treatment of PD patients clinically^21^ (Figure 2(a)). After seven dose administration, high dose (40 mg/kg) and medium dose (20 mg/kg) of NB001also produced a great inhibition of behavioral hyperalgesia, especially high dose (Figure 2(b)). And we found that NB001 at 40 mg/kg (orally) produced a significant reduction of anxiety-related behavior, but not 20 and 10 mg/kg (Figure 1(d)). For motor function, NB001 administration didn’t affect the performance of MPTP-treated mice in RotaRod test and grip strength test (Figure 2(a) and (b)), as well as hanging time and activity time in homecage behavior test (Figure 2(c) and (d)).

### AC1 knockout mice showed decreased pain and anxiety-related behaviors, but not motor function after MPTP administration

**Figure 3.**
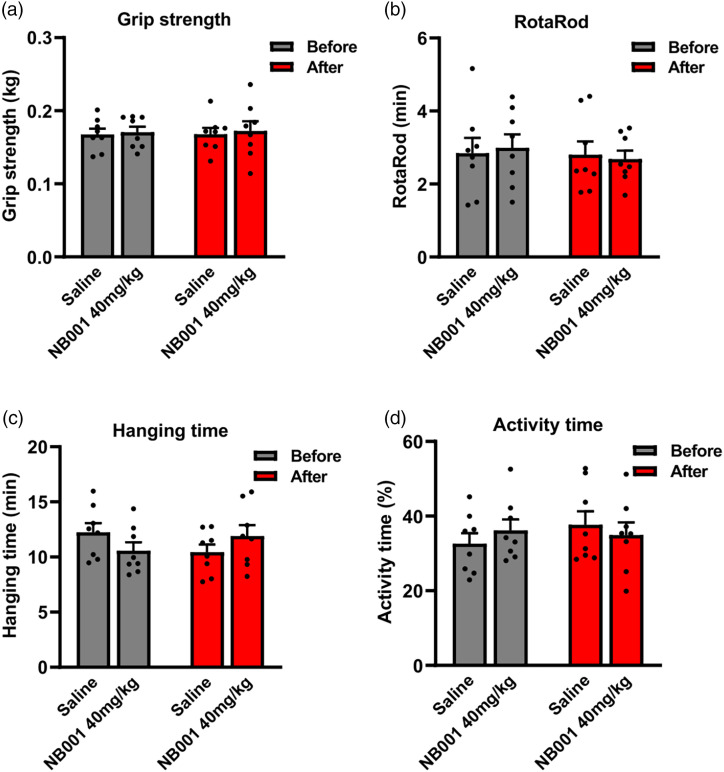
No significant effect of NB001 on motor function. Compared to saline group, oral administration of 40 mg/kg NB001 didn’t affect the performance in grip strength test (a), RotaRod test (b), hanging time (c) and activity time (d) in homecage.

**Figure 4.**
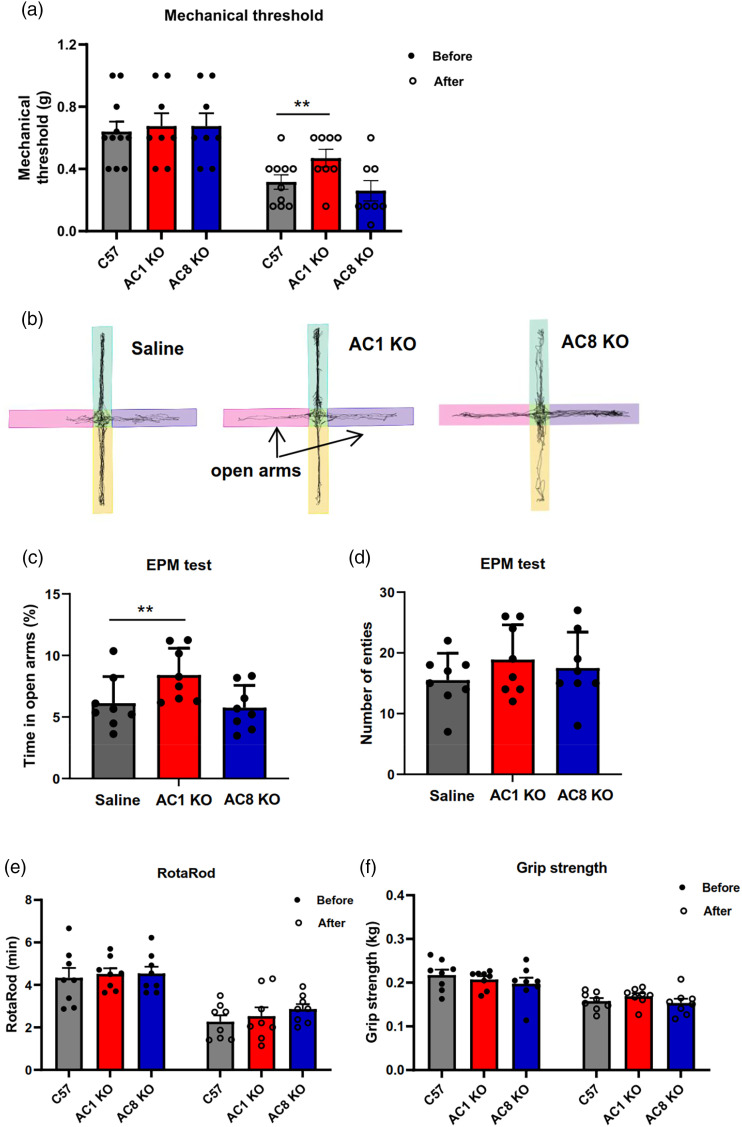
Motor function, pain and anxiety symptoms assessment of MPTP-treated AC1 and AC8 knockout mice. (a–d) Compared to C57 mice, AC1 knockout mice showed decreased pain (a) and anxiety symptoms (c) after MPTP administration, but not AC8 knockout mice, (b) Representative traces showing the movement of mice in EPM, and there was no significant difference of entry number in EPM (d). (e–f) Compared to C57 mice, AC1 and AC8 knockout mice didn’t show significant difference in performance in grip strength test (e) and RotaRod test (f) *p < .05, **p < .01.

(Figure 3) To further verify the effect of AC1 on pain and anxiety symptoms in PD animal mice, we tested pain, anxiety-related behaviors and motor function of MPTP-treated AC1 knockout mice. Considering the possible role of AC8 in anxiety-related behaviors, AC8 knockout mice were also tested. After MPTP administration, AC1 knockout mice showed reduced pain and anxiety response relative to wild-type mice, but not AC8 knockout mice (Figure 4(a) and (c)), and there was no significant difference in the motor function tests (Figure 4(d) and (e)).

## Discussion

**Figure 5.**
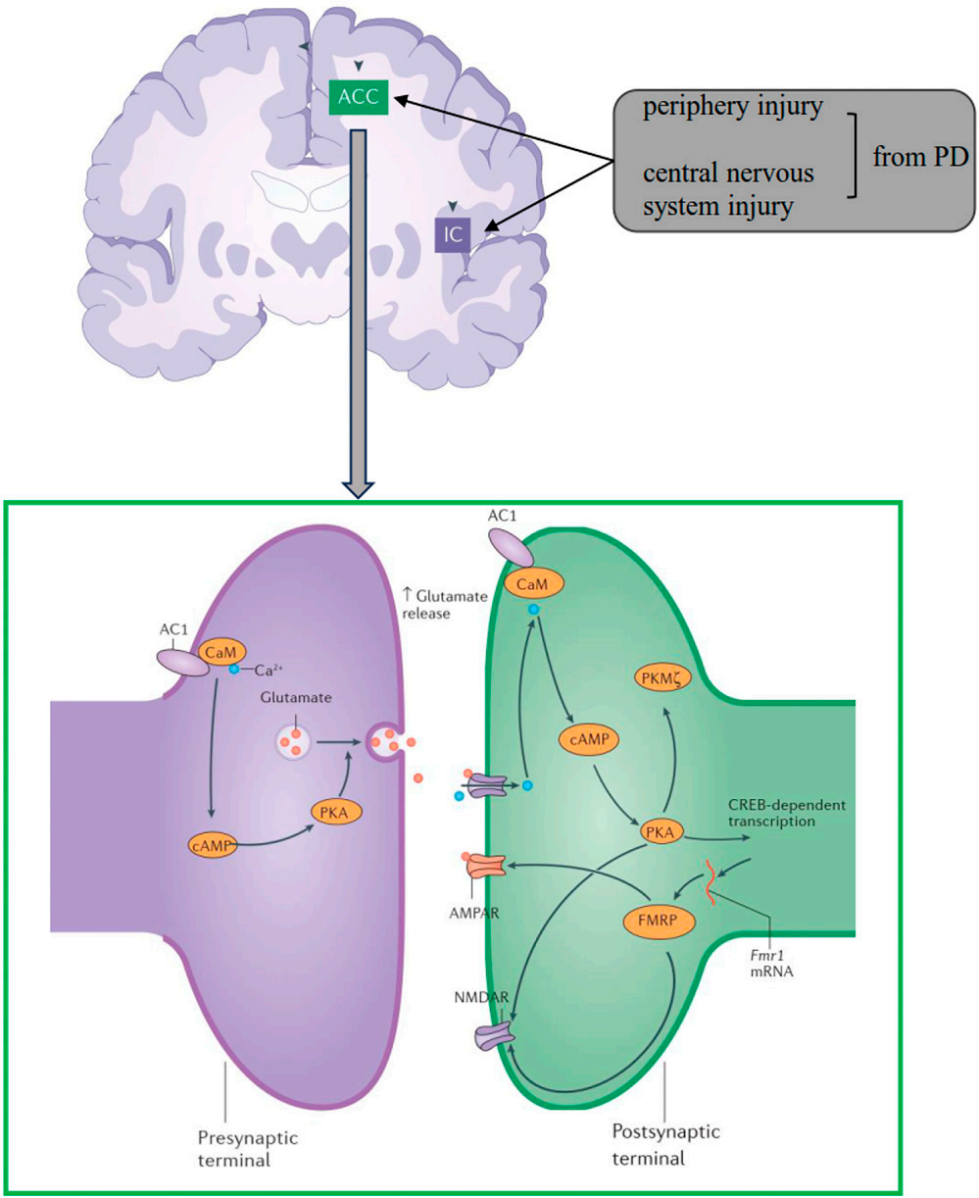
A simplified model for the contribution of AC1 related synaptic plasticity in PD-related pain and anxiety. Synapses in the anterior cingulate cortex undergo long-term presynaptic and postsynaptic changes. At presynaptic sites, glutamate release is increased; at postsynaptic sites, the expression of AMPA receptors (AMPARs) is increased. In addition to postsynaptic increases in AMPAR expression, expression of postsynaptic NMDA receptors (NMDARs), Adenylyl cyclase 1 (AC1) is essential for the presynaptic enhancement of glutamate release and the postsynaptic potentiation of AMPARs and NMDARs. CaM, calmodulin; cAMP, cyclic AMP; FMRP, fragile X mental retardation protein (encoded by Fmr1); PKA, protein kinase A; PKMζ, protein kinase Mζ.

In the present study, we have shown that inhibition of calcium-stimulated AC1 reduced the pain and anxiety behavioral responses of MPTP-treated mice, but not motor function. Our study provides a potential target for clinical treatment of PD-related pain and anxiety symptoms (Figure 5).

### Clinical treatment of Parkinson’s disease-related pain and anxiety

In current clinical treatment of the pain symptom in PD patients, typical dopaminergic compensation approaches and common analgesics are two major pharmacological approaches. Dopaminergic compensation would provide a beneficial effect on the pain resulted from insufficient dopamine, like levodopa, which was reported that the precursor of dopamine reduced pain during the on-state of the pain symptom.^22^ Common analgesics such as opioids (oxycodone, codeine and morphine) has been proved effective to improve different types of pain in PD patients.^23^ However, the addiction and tolerance effects have caused significant medical and social problems.^24^ Therefore, a more selective drug targeting at key molecules in the synaptic mechanism of PD-related pain remains to be found.

Pharmacological treatment of anxiety symptom is common yet problematic for PD patients. PD-related anxiety seems to be associated with dopamine depletion, but it is not clear until now. Indeed, while some studies have found levodopa can improve anxiety symptom, others reported that it may exacerbate anxiety.^25^ Antidepressants and anxiolytics comprise the main approaches for improving anxiety, such as selective serotonin reuptake inhibitors (SSRIs), selective norepinephrine reuptake inhibitors (SNRIs), tricyclics and antipsychotics.^26^ However, use of these medications in PD patients may result in daytime somnolence, cognitive dysfunction, confusion and hallucinations. Clearly, safe, efficacious pharmacologic approaches are needed for PD patients with anxiety symptom.^27^

### AC-cAMP-PKA pathway and dopamine

In dopaminergic system, presynaptically released dopamine interact with postsynaptic dopamine receptor, which is also a kind of G protein-coupled receptor (GPCR). D1-like receptor comprises of D1 and D5 receptors, D2-like receptor is composed of D2, D3, and D4 receptors. D1-like receptors stimulate the G proteins Gα_s_ and Gα_olf_, which are positively coupled to ACs, leading to the production of cyclic adenosine monophosphate (cAMP) and the activation of protein kinase A (PKA). By contrast, D2-like receptors activate Gα_i_ and Gα_o_ proteins, which inhibit AC and limit PKA activation. The AC-cAMP-PKA pathway in the ACC is activated in chronic pain.^28^ Therefore, we can infer that, in the condition of PD, when the concentration of dopamine decreased due to the neurodegeneration of midbrain dopaminergic neurons, D2-mediated inhibition might be alleviated, thus the AC-cAMP-PKA pathway is disinhibited. AC1 and AC8 are two major ACs subtypes, which may be involved in the mechanism between AC-cAMP-PKA pathway and dopamine. Compared with AC8, AC1 is more sensitive to Ca^2+^ increases.

### AC1, a potential safe drug target

In the original discovery of AC1 in pain research, AC1 KO mice didn’t show lack of phenotype in learning-related LTP and behavioral memory.^29^ What’s more, cognitive, emotional, motor functions, and acute sensory functions are intact in AC1 KO mice, which made it more attractive for candidates of chronic pain.^29^ Through rational drug design and chemical screening, Wang et al. identified a lead candidate AC1 inhibitor, NB001, which is relatively selective for AC1 over other AC isoforms.^30^ Using a variety of behavioral tests and toxicity studies, Wang et al. showed that NB001, when administered intraperitoneally or orally, had an analgesic effect in animal models of neuropathic pain, without any apparent side effects.^30^ NB001 also produced a significant analgesic effect in both acute persistent and chronic inflammatory muscle pain.^31^ Zhang et al. reported that NB001 produced inhibition of injury-induced behavioral anxiety and spontaneous pain in a visceral pain model of IBS,^32^ and AC1 was also upregulated in this model.^13^ Besides animal model studies, human-used NB001 (hNB001) is found to be safe in healthy human subjects.^33^

AC1 is required for the induction of cortical LTP, including ACC and IC. Furthermore, protein kinase Mζ (PKMζ), which is required for maintenance for LTP in the ACC, is also regulated by AC activity. In animal models with injury, AC1 is required for injury induced excitatory transmission, including postsynaptic and presynaptic changes. This enhancement is mainly mediated by AMPA receptors and NMDA receptors. In addition to its contribution to the ACC, similar results have been reported in the IC recently.^21,22^ For example, AC1 contributes to the upregulation of synaptic NMDARs after nerve injury in the IC.

Previous studies have clearly demonstrated that AC1 is a good candidate to block behavioral sensitization and anxiety in animal models with few or no side effects. This study discovered the analgesic and anxiolytic effects of NB001 in PD model mice, and future studies are needed to clarify the molecular and synaptic mechanisms. In summary, we provide strong evidence that NB001 is a potential drug for the treatment of pain and anxiety symptoms in PD patients.
